# Multifactorial Analysis of Endodontic Microsurgery Using Finite Element Models

**DOI:** 10.3390/jpm12061012

**Published:** 2022-06-20

**Authors:** Raphael Richert, Jean-Christophe Farges, Jean-Christophe Maurin, Jérôme Molimard, Philippe Boisse, Maxime Ducret

**Affiliations:** 1 Hospices Civils de Lyon, PAM Odontologie, 69007 Lyon, France; jean-christophe.farges@univ-lyon1.fr (J.-C.F.); jean-christophe.maurin@univ-lyon1.fr (J.-C.M.); maxime.ducret@univ-lyon1.fr (M.D.); 2 Laboratoire de Mécanique des Contacts et Structures, UMR 5259 CNRS/INSA Lyon\Université de Lyon, 69100 Villeurbanne, France; philippe.boisse@insa-lyon.fr; 3 Laboratoire de Biologie Tissulaire et Ingénierie Thérapeutique, UMR 5305 CNRS\Université Claude Bernard Lyon 1, 69008 Lyon, France; 4 Mines Saint-Etienne, Centre CIS, INSERM, U 1059 Sainbiose, Université de Lyon\Université Jean Monnet, 42000 Saint-Etienne, France; molimard@emse.fr

**Keywords:** finite element analysis, endodontics, decision aid

## Abstract

Background: The present study aimed to classify the relative contributions of four biomechanical factors—the root-end filling *material*, the apical *preparation*, the root *resection length*, and the *bone height*—on the root stresses of the resected premolar. Methods: A design of experiments approach based on a defined subset of factor combinations was conducted to calculate the influence of each factor and their interactions. Sixteen finite element models were created and analyzed using the von Mises stress criterion. The robustness of the design of experiments was evaluated with nine supplementary models. Results: The current study showed that the factors *preparation* and *bone height* had a high influence on root stresses. However, it also revealed that nearly half of the biomechanical impact was missed without considering interactions between factors, particularly between resection and *preparation*. Conclusions: Design of experiments appears to be a valuable strategy to classify the contributions of biomechanical factors related to endodontics. Imagining all possible interactions and their clinical impact is difficult and can require relying on one’s own experience. This study proposed a statistical method to quantify the mechanical risk when planning apicoectomy. A perspective could be to integrate the equation defined herein in future software to support decision-making.

## 1. Introduction

Endodontic microsurgery (EMS) is a well-established strategy for the endodontic treatment of non-healing apical periodontitis [1]. During the past decades, the success rate has largely been improved with the use of microscopes, ultrasonic microtips for root-end cavity *preparation*, and biocompatible root-end filling materials [1]. However, this success rate and the clinical indications of EMS are now limited by the mechanical resistance of the tooth [1,2]. EMS is indeed often proposed after previous endodontic treatments in cases of severely damaged teeth often bearing a post, but this procedure also leads to a reduction in the crown-to-root ratio, leading to a higher risk of vertical root fracture [1,2]. Vertical root fracture leads to inflammation in the supporting tissues. Periodontal attachment loss has been reported to be a highly predictive factor of EMS failure [3]. However, the mechanisms leading to this vertical root fracture remain unclear.

Meta-analyses have been conducted to identify the prognostic factors for EMS [3,4,5]. It appears that different tooth-related factors, such as the tooth being a premolar or the presence of a post, significantly increase the risk of vertical root fracture [2]. However, the precise influence of numerous other treatment-related factors, such as retro-*preparation* or *retro-filling material*, remains debated [5,6]. Similarly, bone height and periodontal lesions have also been reported to be predictive of success, but the biomechanical impact also depends on other factors, such as the *resection length* [3,7]. A better understanding of the key mechanisms leading to vertical root fracture is of great importance, as biased decision-making could lead to shortcuts based on the clinician’s experience rather than scientific evidence [2]. Finite element (FE) analysis has been commonly used to investigate the biomechanical properties of resected teeth and damaged premolars [6,7,8,9,10]. Among the different prognostic factors of EMS, the biomechanical factors frequently evaluated by FE studies are *retro-filling material*, retro-*preparation*, root *resection length*, and *bone height* [6,7,8,9]. It appears that the biomechanics related to EMS are highly multifactorial [6,7,8,9]. Consequently, the relative contribution of each factor to the overall outcome remains unclear, and published studies studying the interactions between factors are limited. Recently, a statistical approach based on a subset of defined factor combinations and FE models was proposed to understand multifactorial situations in adhesive dentistry and enable the most influential factors to be defined [10]. Our hypothesis was that a similar strategy could be used to classify the relative contributions of four biomechanical factors that influence the root stresses of a resected premolar.

The current study aimed to define an equation representative of the biomechanics of a resected premolar with the perspective of adding this to decision support software.

## 2. Materials and Methods

### 2.1. Premolar Model

An intact human maxillary premolar extracted for orthodontic reasons was scanned using cone-beam computed tomography (Planmeca ProMax 3D, Helsinki, Finland) operating at 120 kV, 100 mAs, with a slice thickness of 75 μm. Written informed consent was obtained from the patient who provided the premolar. The different anatomical structures were segmented using a semi-automatic procedure [11]. The segmented 3D image was modified to model a post, a crown, and the apicoectomy. The alveolar bone and a 0.2 mm-thick periodontal ligament were simulated around the root [12]. The segmented 3D image was then meshed using quadratic tetrahedral elements after a convergence test. All dental materials were assumed to be homogeneous and linearly elastic except for the periodontal ligament, which was assumed to be hyper-elastic. The attributed material properties were referenced from the literature (Table 1) [13].

**Figure 1:**
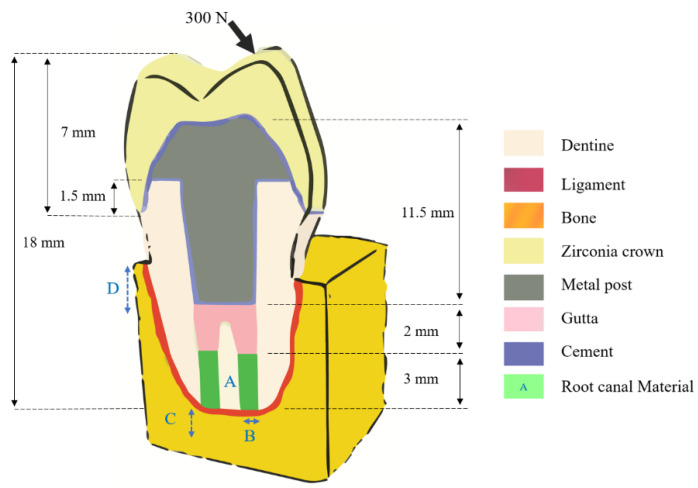
Representative schema of the resected tooth with A the root end-filling material, B the retro preparation, C the resection level, and D the bone height.

There was a perfect bonding between each component (16), and a static oblique load of 300 N was applied to the vestibular cuspid of the crown to simulate masticatory forces (Figure 1). The nodes of the lateral faces of the cortical bone were constrained to prevent displacement. The FEA was conducted on the Abaqus software 6.14 (Dassault Systèmes, Vélizy-Villacoublay, France) to calculate the von Mises root stresses of the resected premolar.

### 2.2. Biomechanical Factors

A design of experiments (DOE) approach was then used to compare the influence of four major factors on root stress: A—the Young’s modulus of the root-end filling *material*, B—the apical *preparation*, C—the root *resection length*, and D—the *bone height*. The following equation was used to model the upper decile of von Mises root stresses, S, as a linear function of the input factor values and interactions between them. von Mises stress is a scalar value combining the three principal stresses into an equivalent stress that can be used to judge the failure condition of the material [13].

(1) $$S=S_{0}+\overset{4}{\underset{i=1}{\sum }}a_{i}S_{i}+\overset{4}{\underset{\begin{array}{c} i=1\\ j=2\\ i<j \end{array}}{\sum }}a_{ij}S_{i}S_{j}+\overset{4}{\underset{\begin{array}{c} i=1\\ j=2\\ k=3\\ i<j<k \end{array}}{\sum }}a_{ijk}S_{i}S_{j}S_{k}+a_{1234}S_{1}S_{2}S_{3}S_{4}\;$$

where a_0_ is the mean overall response, [a_1_,a_2_,a_3_,a_4_] are the main effects of factors [S_1_,S_2_,S_3_,S_4_] on the response; [a_12_,a_23_,a_24,_a_34_,a_13_,a_14_] are the interactions between factors of order 1; [a_123_,a_234_,a_124_,a_134_] are the interactions of order 2; and a_1234_ are the interactions of order 3. Two extreme levels were defined for each factor and were encoded as [−1; +1]. In this way, the effect (resp. interaction) ai directly represents the von Mises stress variation when the factor (resp. interaction) varies from the mean value (encoded −1) to the upper bound (encoded +1). Regardless of the physical nature of each factor, it is possible to directly compare their effects ai on the von Mises stress. The Young’s modulus of the polymer-reinforced zinc oxide–eugenol cement and the Mineral Trioxide Aggregate (MTA), respectively, were used for −1 and +1 levels of the retro filling *material* [9,14] (Table 2).

An orthogonal array was created to model the 16 possible combinations depending on the level of each factor. A static explicit analysis was conducted to calculate the highest von Mises stresses for the 16 FE models. The FE models M1 to M8 report the stress values for a low level of bone, and models M9 to M16 report the values for a high level of bone (Table 3). The coefficients of Equation (1) were then defined by inversion of the orthogonal matrix, and the 10 most influential coefficients of Equation (1) were represented in descending order on a Pareto chart.

### 2.3. Design of Experiments Validation

The robustness of the DOE was evaluated using 9 supplementary models defined by levels [0; +1] [−1; 0] for each factor. An analysis of variance (ANOVA) was then used to validate the DOE validation [10]. The mean square error (MSE), mean square regression (MSR), and the ratio F_value_ were calculated as shown below. The validity of the model was controlled using a Fisher law F_test_ with an 80% confidence interval (CI) and under the null hypothesis *H*_0_.

(2) $$\{\begin{array}{c} F_{value}=\frac{MSR}{MSE}=\frac{\left(n-p\right)\sum_{1}^{n}{\left(\hat{S_{l}}-\overset{´}{S}\right)}^{2}}{\left(p-1\right)\sum_{1}^{n}{\left(S_{l}-\hat{S_{l}}\right)}^{2}}\\ H_{0}\;rejected\;if\;F>F_{test}\left(p-1,n-p\right), \end{array}$$

where $\hat{S_{l}}$ is the response calculated by the FE model, *S_l_* is the simulated response calculated by Equation (1), $\overset{´}{S}$ is the mean response, and *n* and *p* represent the total number of models and the number of parameters, respectively.

## 3. Results

The FE models with a low *bone height* had higher root stresses than those with a high *bone height*. For high levels of Young’s modulus *material* and *resection length*, FE models with a high level of *preparation* had lower root stresses than those with a low level of *preparation*, but the contrary was found for a low level of Young’s modulus (Table 3).

**Figure 2:**
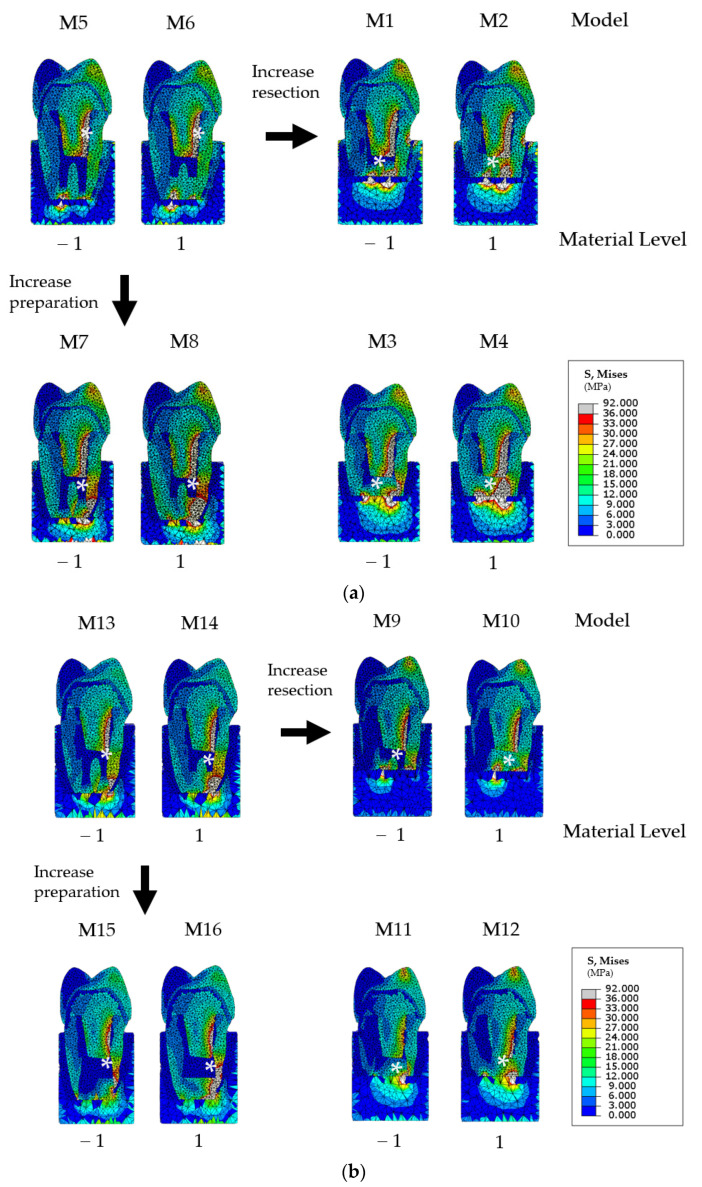
Von Mises root stresses represented by a color diagram from blue (low values) to red (high values); (**a**) simulations M1 to M8 are related to finite element (FE) models with a low bone height, and (**b**) simulations M9 to M16 are related to finite element (FE) models with a low bone height. The area of highest stress is indicated by a white asterisk.

For all FE models, high stresses were located on the vestibular side of the post. For FE models with a high level of *resection length*, high stresses were also present on the apical part of the root. For FE models combining a low level of resection and a high *preparation*, high stresses were present on the apical as well as the vestibular parts of the root (Figure 2).

**Figure 3:**
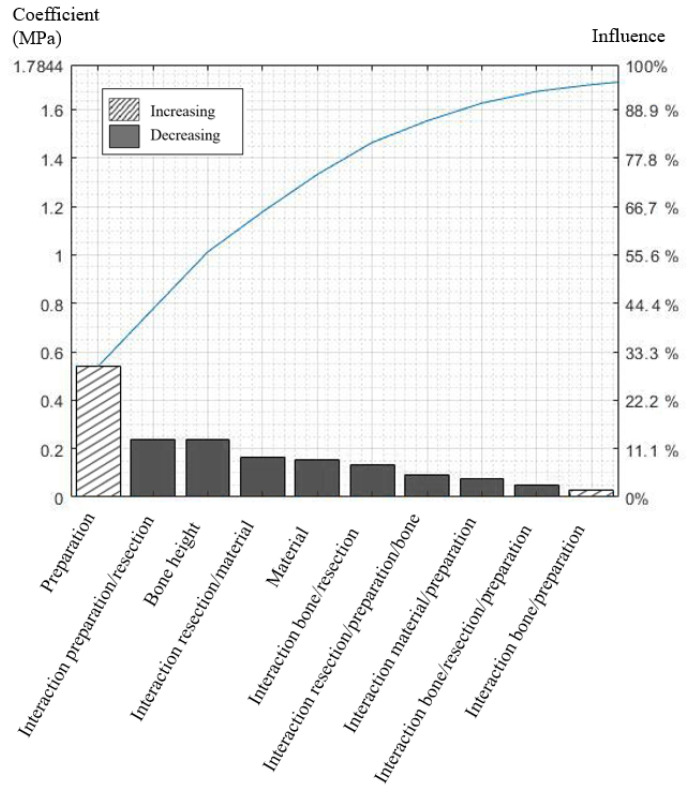
Pareto chart representing the absolute values of the 10 strongest effects and interactions in descending order as bars and the cumulative total as a line. The difference between 0 and 100% represents the variation between lowest and highest stress.

A high level of *bone height* or Young’s modulus *material* reduced root stresses, whereas a high *preparation* or *resection* increased it. The coefficient for the interaction *preparation*/*resection* was high and reduced root stress. This indicates that *resection length* has little effect on root stress for a low level of *preparation*, and, conversely, *resection length* has a great effect on root stress for a high level of *preparation*. The cumulative total value of effects indicated that 48% of von Mises stresses could not be modeled without interaction factors and that at least 10 of the 16 coefficients need to be considered to model 95% of the total effects (Figure 3). The interactions of order 1 enabled the modeling of 90% of the total effects (Supplementary Material, Table S1). The model was considered valid because the F_value_ = 21.42 was superior to F_test_ = 1.7 (80% CI).

## 4. Discussion

The success rate of EMS has largely been improved during the past decades, but it is still limited by the biomechanical resistance of the weakened tooth (2–4). The equation defined herein indicates that the most influential factors are the *preparation* and *bone height* but that it is also important to consider the interactions that explain nearly half of the root stress.

The current study confirmed that a high *bone height* had a high impact on reducing root stresses [6,15]. Herein, the bone height increased the root stress by more than 20% on average when it was low, but this factor has been reported to be even more important in cases of overjet (90% increase) [6,15]. However, in cases of low *bone height*, other biomechanical factors, such as the retro-filling *material*, need to be considered to preserve the tooth. The influence of the *material* on the success rate at one and five years is still debated [1,16,17,18,19,20]. Nevertheless, in the present study, FE models with a high Young’s modulus *material* (such as MTA) had lower stresses than those with a low Young’s modulus material (such as polymer-reinforced zinc oxide–eugenol cement). This result could be explained by the Young’s modulus of MTA being similar to dentine, whereas it is 10 times lower for polymer-reinforced zinc oxide–eugenol cement [21]. This *material* factor has yet to be analyzed specifically in cases of low *bone height* when root stresses are increased. However, it was reported that immature teeth filled with MTA had higher fracture resistance in vitro than those filled with gutta-percha [21,22,23]. Recently, new silicate-based materials, such as Biodentine (Septodont, Saint-Maur-des-Fossés, France), were reported to have superior mechanical properties, but this material still presents limitations due to a lack of radiopacity [5]. The equation defined herein offers the possibility to virtually investigate the behavior of newly developed materials before setting up in vitro investigations.

*Resection length* is a frequently investigated factor [6,15]. Using MTA, a reduction in the *resection length* led to a 3 to 10 MPa decrease in the maximal root stress, which is in accordance with previous FE studies [6,7]. This also confirms that the smallest *resection* level of 3 mm should be recommended in clinical practice and that this factor has a negligible mechanical effect compared with other factors, such as bone *height* [6,15]. However, the influence of the retro *preparation* was not evaluated in these studies, whereas herein, it was the most influential factor. High retro *preparation* could lead to numerous procedural errors, such as over-enlarged preparation or preparation being off-angle from the root canal space [24], which could result in reduced biomechanical properties. The converse effects were also found herein for certain combinations of factors, suggesting that the effect of the *resection length* could be influenced by *preparation*. Considering low *resection length* levels, the results of the present study confirm that a high *preparation* level decreases the root stresses, as previously reported by Kim et al. [8,9]. However, for other combinations of *resection length,* a high *preparation* level increased the root stresses, confirming the presence of interactions. The high positive interaction between *resection* and *preparation* suggests that a high *resection* is indicated for a *high preparation*. This result can be explained by the fact that a high *preparation* level induces very thin dentin walls prone to fracture, but this would have been attenuated by a higher level of *resection* [25]. Considering all coefficients of the equation, the DOE suggested that nearly half of the biomechanical impact is missed without considering interactions between factors, which is of great importance because these interactions should be present in all situations encountered in EMS. Imagining all possible interactions between parameters and their clinical impact is difficult and can require relying on one’s own experience rather than scientific evidence [26]. Interestingly, the results of the present study indicate that interactions of order 1 are sufficient to model the biomechanics of the resected tooth, and a perspective of this work will be to integrate the current equation into decision support software to assist endodontists during the decision-making process [27].

The present study does, however, have several limitations that should be considered when interpreting the findings. For instance, von Mises stress was used as the failure criterion under the assumption that dentin could sustain plastic deformation before fracture [28]. However, other failure criteria that neglect plasticity could have been used, such as the maximum principal stress [13], which could have changed the results of the DOE. Furthermore, the development of adhesive dentistry and more conservative fiber posts has considerably changed the tooth restoration, and further investigations now appear to be required to evaluate the influence of these new strategies on EMS [29,30]. Moreover, EMS also aims to treat persisting apical periodontitis due to extra-radicular infection without the presence of a post. In these cases, the volume and location of the lesion could alter the tooth deformation under masticatory loads, emphasizing the need to better understand the biomechanical aspects of bone grafting [8]. Another general point is that more than 20 other factors, including patient-specific root canal anatomy and different occlusions, have been reported as prognostic factors in the success of EMS [1]; therefore, numerous simulations are required to complete the biomechanical analysis of the resected tooth. These considerations emphasize the need to automate and personalize the FEA using validated parameters [13,29]. Finally, the numerical method developed herein should not lead clinicians to reduce the outcome purely to the biomechanical aspect, as other key aspects, such as the use of an operating microscope and ultrasonic instruments, are essential to reveal anatomical details and enhance the cleaning of the root canal space [31].

## 5. Conclusions

The current work proposed an original equation combining the influence of the four main biomechanical factors in apicectomy. The results suggest that nearly half of the biomechanical impact is missed without considering interactions between factors. In such a multifactorial situation, a decision support software could be of benefit to clinicians in planning and personalizing endodontic microsurgery.

## Figures and Tables

**Table 1:** Material properties [13].

Material	Model
Dentine	Linear elastic isotropic E = 18,600 MPa, ν = 0.31
Ligament	Hyper-elastic Ogden 1 μ = 0.12 MPa, α= 20.9 MPa, D = 10
Trabecular bone	Linear elastic isotropic E = 1300 MPa, ν = 0.3
Cortical bone	Linear elastic isotropic E = 13,000 MPa, ν = 0.3
Zirconia Crown	Linear elastic isotropic E = 190,000 MPa, ν = 0.33
Metal Post	Linear elastic isotropic E = 190,000 MPa, ν = 0.33
Gutta	Linear elastic isotropic E = 69 MPa, ν = 0.45
Cement	Linear elastic isotropic E = 3000 MPa, ν = 0.3
Root canal Material 1	Linear elastic isotropic E = 1000 MPa, ν = 0.3
Root canal Material 2	Linear elastic isotropic E = 22,000 MPa, ν = 0.3

**Table 2:** Investigated parameters and extreme levels assigned for each one.

Parameter	Low Level (−)	High Level (+)
A—Material	1000 MPa	22,000 MPa
B—Preparation	1.5 mm	2.2 mm
C—Resection length	3 mm	6 mm
D—Bone height	−2 mm	0 mm

**Table 3:** Upper decile von Mises stress obtained for each finite element model and associated orthogonal matrix (−1/+1 indicate the level assigned to each factor).

Low Bone Height						High Bone Height					
**Model**	Material	Preparation	Resection	Bone	VMS ^1^	**Model**	Material	Preparation	Resection	Bone	VMS ^1^
**M1**	−1	−1	+1	−1	1.76 MPa	**M9**	−1	−1	+1	+1	1.40 MPa
**M2**	+1	−1	+1	−1	1.75 MPa	**M10**	+1	−1	+1	+1	1.39 MPa
**M3**	−1	+1	+1	−1	2.59 MPa	**M11**	−1	+1	+1	+1	1.88 MPa
**M4**	+1	+1	+1	−1	2.26 MPa	**M12**	+1	+1	+1	+1	1.58 MPa
**M5**	−1	−1	−1	−1	3.14 MPa	**M13**	−1	−1	−1	+1	3.06 MPa
**M6**	+1	−1	−1	−1	3.17 MPa	**M14**	+1	−1	−1	+1	3.14 MPa
**M7**	−1	+1	−1	−1	3.62 MPa	**M15**	−1	+1	−1	+1	2.71 MPa
**M8**	+1	+1	−1	−1	2.52 MPa	**M16**	+1	+1	−1	+1	1.88 MPa

^1^ VMS: von Mises Stress.

## Data Availability

The data presented in this study are available on request from the corresponding author.

## Supplementary Materials

The following supporting information can be downloaded at: https://www.mdpi.com/article/10.3390/jpm12061012/s1, Table S1: Mean and coefficient for each contribution in Equation (1) (own influence and interactions).
