# Supplementary material for: Multifactorial Analysis of Endodontic Microsurgery Using Finite Element Models
Source: J Pers Med. 2022 Jun 20;12(6):1012. doi: 10.3390/jpm12061012 (PMC9224708; doi:10.3390/jpm12061012)
Supplement: Supplementary file 1 [file jpm-12-01012-s001.zip › jpm-1748395-supplementary.pdf]

Supplementary material

Table S1 Mean and coefficient for each contribution in equation (1) (own influence and interactions).

	**Coefficient**	**Coefficient (MPa)**	**Influence (%)**
Mean	Mean	2.365625	
Own influence parameter	Material	-0.235625	13.204904
	Preparation	0.539375	30.227671
	Resection	0.014375	0.805604
	Bone	-0.154375	8.651489
Interactions between parameters order 1	Interaction material/preparation	0.028125	1.576182
	Interaction material/resection	-0.131875	7.390543
	Interaction material/bone	0.021875	1.225919
	Interaction preparation/resection	-0.236875	13.274956
	Interaction preparation/bone	-0.073125	4.098074
	Interaction resection/bone	-0.165625	9.281961
Interactions between parameters order 2	Interaction material/preparation/resection	-0.048125	2.697023
	Interaction material/preparation/bone	0.018125	1.015762
	Interaction material/resection/bone	0.015625	0.875657
	Interaction preparation/resection/bone	-0.089375	5.008757
Interactions between parameters order 3	Interaction material/preparation/resection/bone	0.011875	0.665499
